# Analysis of influencing factors and paths of social frailty in older adult patients with ischemic stroke: a cross-sectional study

**DOI:** 10.3389/fpubh.2025.1678028

**Published:** 2025-10-20

**Authors:** Yina Liu, Jing Zhao, Jing Feng, Lijun Cui, Qingwen Long, Ying Yang, Yang Hu, Yiting Yin, Li Li

**Affiliations:** ^1^School of Nursing, North Sichuan Medical College, Nanchong, Sichuan, China; ^2^Department of Nursing, Daying County People's Hospital, Suining, Sichuan, China; ^3^Department of Blood Transfusion, Affiliated Hospital of North Sichuan Medical College, Nanchong, Sichuan, China

**Keywords:** older adult, ischemic stroke, social frailty, influencing factors, structural equation model

## Abstract

**Objective:**

Analysis of influencing factors and action pathways of social frailty in older adult patients with ischemic stroke.

**Methods:**

A cross-sectional study was conducted among older adult inpatients with ischemic stroke in the Department of Neurology of two Grade A tertiary hospitals in Sichuan Province, China, from January to May 2025. Multiple stepwise linear regression analysis was used to analyze the influencing factors of social frailty in older adult patients with ischemic stroke, and structural equation modeling was employed to conduct path analysis on these influencing factors.

**Results:**

A total of 437 older adult patients with ischemic stroke completed the study. The prevalence of social frailty in older adult ischemic stroke patients was 38%. Regression analysis showed that activities of daily living, sleep quality, depression, social support, employee medical insurance, language function impairment and stroke recurrence are influencing factors of social frailty levels in older adult patients with ischemic stroke (*p* < 0.05). The structural equation model revealed that in the biological dimension, the direct effect of ADL on social frailty was significant (*β* = −0.154, *p* = 0.002), accounting for 16% of the total direct effects, while its indirect effect through social support was also significant (*β* = −0.061, *p* = 0.003, 95% CI: −0.114 to −0.019), representing 28% of the total effect in the ADL-social frailty pathway; the direct effect of sleep quality on social frailty (*β* = 0.057, *p* = 0.282) and the indirect effect via social support (*β* = 0.014, *p* = 0.546, 95% CI: −0.032 to 0.062) were not significant. In the psychological dimension, depression had a significant direct effect on social frailty (*β* = 0.390, *p* < 0.001), accounting for 40% of the total direct effects, along with a significant indirect effect via social support (*β* = 0.113, *p* < 0.001, 95% CI: 0.063–0.181), representing 22% of the total effect in the depression-social frailty pathway. In the social dimension, social support showed a significant direct effect on social frailty (*β* = −0.373, *p* < 0.001), representing 38% of the total direct effects.

**Conclusion:**

This study found that the prevalence of social frailty in older adult patients with ischemic stroke was 38%, and it is closely associated with health insurance type, stroke recurrence, language function, activities of daily living, depression, and social support. These findings provide a reference for clinical practitioners to design targeted interventions.

## 1 Introduction

According to the Global Burden of Disease, Injuries, and Risk Factors Study, stroke is the third leading cause of death globally and ranks fourth in terms of disability-adjusted life years (DALYs), with ischemic stroke accounting for 65.3% of all stroke cases (1). In China, despite the implementation of nationwide screening and intervention programs targeting high-risk populations, ischemic stroke remains highly prevalent, with both its incidence and prevalence continuing to rise (2). Aging is a major predictive factor for the incidence and mortality of ischemic stroke (3). With the rapid development of an aging society in China, the situation regarding older adult patients with ischemic stroke in China may become more severe in the future. Against this backdrop, how to improve the quality of life of older adult patients with ischemic stroke has become an important public health issue that urgently needs to be addressed.

Social frailty (SF), a common geriatric syndrome, is defined as an individual’s inadequacy in social resources, behaviors, activities, and self-management capabilities to meet basic needs (4). It includes, but is not limited to, social alienation, weakened social roles, and feelings of loneliness (5). In older adult ischemic stroke patients, social frailty-related characteristics are widely observed across multiple dimensions. According to Kam and Choo (6), among 160 stroke patients, 30.6% experienced feelings of loneliness, while only 28.1% reported high satisfaction with their social networks. Wu et al. (7) found a relatively high level of social isolation in stroke patients. Chen and Che (8) emphasized that these patients often face deficits in self-management capabilities. Additionally, research showed a decline in their social participation in areas such as interpersonal communication, social activities, and work (9). These changes render older adult ischemic stroke patients more susceptible to social frailty.

Although numerous studies have demonstrated that social frailty is associated with adverse outcomes including disability, rehospitalization, and even mortality, and that it significantly impairs patients’ quality of life (10–12), research on social frailty among older adult patients with ischemic stroke remains extremely limited in China. Therefore, there is an urgent need to identify the potential influencing factors and pathways underlying social frailty in this patient population, to facilitate the early identification of high-risk groups, the development of intervention strategies, and the enhancement of patients’ quality of life in the social domain.

Many studies have found that social frailty is associated with activities of daily living (ADL), sleep quality, depression, and social support. For example, the study by Li et al. (13) used the Least Absolute Shrinkage and Selection Operator and random forest to analyze the influencing factors of social frailty in older adult individuals living with HIV/AIDS. The results showed that ADL, sleep disorders, depression, and social support were important factors. A study from Iran reported that depression increases the risk of social frailty in older adults (14). Liu et al. (15) adopted a latent class growth model to explore the predictors of social frailty in stroke patients. The findings revealed that social support and ADL were significant protective factors against social frailty, with higher levels of both associated with lower social frailty risks; however, the study did not examine the interactive pathways among multiple factors and consequently could not elucidate the complex mechanisms underlying the relationships between these influencing factors. Qiao et al. (16) analyzed influencing factors of social frailty in older adults with minor ischemic stroke using a random forest model. The results indicated that ADL was a significant influencing factor; however, the study did not include depression, sleep quality, or social support as potential predictors. While these studies have contributed to identifying key factors, their approaches preclude an understanding of the integrated pathways. These gaps leave the bio-psycho-social multipath mechanisms underlying social frailty in older adults with ischemic stroke still unclear.

**Figure 1:**
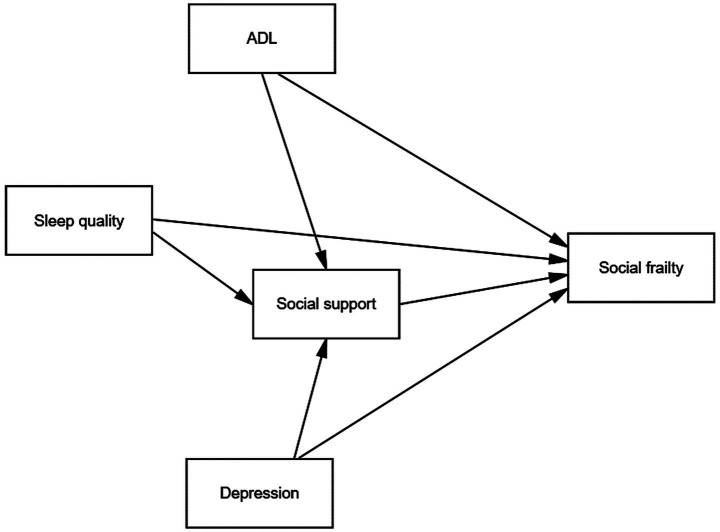
The theoretical model of research hypotheses. - Biological factors: H1a: ADL may directly affect social frailty. H1b: ADL may indirectly affect social frailty through social support. H2a: Sleep quality may directly affect social frailty. H2b: Sleep quality may indirectly affect social frailty through social support. - Psychological factors: H3a: Depression may directly affect social frailty. H3b: Depression may indirectly affect social frailty through social support. - Social factors: H4: Social support may directly affect social frailty.

To address these limitations and provide a theoretical foundation for exploring these integrated pathways, it is necessary to move beyond examining independent factor effects and delve into the complex interactions among variables. To this end, we draw upon two theoretical frameworks. First, the bio-psycho-social medical model emphasizes that an individual’s health status is the result of the synergistic action of biological, psychological, and social factors, rather than the independent influence of each factor (17). Furthermore, the social support buffer system theory highlights that social support can buffer part of the harm when individuals are exposed to stressors (18). Therefore, based on the bio-psycho-social medical model and the social support buffer system theory, this study constructs a theoretical model (see Figure 1) and proposes the following research hypotheses:

Factors (for older adult patients with ischemic stroke):

- Biological factors:

*H1a*: ADL may directly affect social frailty.

*H1b*: ADL may indirectly affect social frailty through social support.

*H2a*: Sleep quality may directly affect social frailty.

*H2b*: Sleep quality may indirectly affect social frailty through social support.

- Psychological factors:

*H3a*: Depression may directly affect social frailty.

*H3b*: Depression may indirectly affect social frailty through social support.

- Social factors:

*H4*: Social support may directly affect social frailty.

This study uses structural equation modeling to examine the interrelationships among influencing factors, their action pathways on social frailty, and the magnitude of effects within each pathway. It should be particularly noted that structural equation modeling enables the simultaneous testing of both direct and mediating effects—a methodological advantage that effectively addresses previous studies’ limitations in examining complex multifactorial mechanisms. By integrating multiple determinants, structural equation models uncover complex interactions, improving our understanding of social frailty. The identified pathways help clinicians develop targeted interventions. By addressing the key determinants identified in this study, these strategies can reduce social frailty and improve patients’ social function and quality of life.

## 2 Materials and methods

### 2.1 Participants

This study adopted a cross-sectional survey design and used a convenience sampling method to investigate older adult patients with ischemic stroke hospitalized in the neurology departments of two general hospitals in China from January to May 2025.

The inclusion criteria were as follows: (1) age ≥60 years; (2) Meeting the diagnostic key points as defined by the Diagnostic Criteria of Cerebrovascular Diseases in China (Version 2019) (19) and being diagnosed with ischemic stroke by an attending physician; (3) stable condition.

The exclusion criteria were as follows: (1) being in the acute phase of ischemic stroke; (2) having severe mental illness, unable to cooperate with the survey; (3) having severe aphasia and being unable to complete the study by any means, including self-administered questionnaires, body gestures, facial expressions, or other auxiliary communication methods; (4) having impaired consciousness; (5) having cognitive impairment.

### 2.2 Calculation of sample size

According to the formula for calculating sample size in cross-sectional studies *n =* [(*μ_1 − α/2_* × *σ*)/*δ*]^2^ (20). Among these, “*n*” denotes the estimated sample size, “*μ_1 − α/2_*” represents the critical value from the standard normal distribution, “*σ*” indicates the population standard deviation of the outcome measure, and “*δ*” corresponds to the margin of error. With a two-tailed test *α* = 0.05, *μ_1–α/2_* = 1.96. According to the results of the preliminary pre-survey of 40 cases, the standard deviation of the score of social frailty in older adult ischemic stroke patients was calculated as *σ* = 1.35, and the allowable error *δ* was 10% of the standard deviation *σ*, *δ* = 0.135. The sample size *n* = 385 was calculated, and an additional 10% of invalid questionnaires were added, resulting in a final sample size of *n* = 424 cases.

### 2.3 Ethical considerations

This study was approved by the Ethics Committee of the Affiliated Hospital of North Sichuan Medical College (2025ER48-1). All subjects signed the informed consent form. All procedures conformed to the principles outlined in the Declaration of Helsinki.

### 2.4 Data collection and quality control

Prior to data collection, consistency training was conducted for the two survey investigators. The investigators used a standardized protocol to explain the purpose and significance of the study to the patients. After obtaining informed consent, they administered paper-based questionnaires in a quiet room. The assessment duration was approximately 20 min. For patients unable to complete the questionnaires independently, the investigators objectively rephrased the questionnaire items and recorded the patients’ responses accordingly, with the evaluation process lasting approximately 30 min. Investigators checked the completeness of the questionnaires on-site and supplemented any missing items immediately.

A total of 450 questionnaires were distributed; however, nine patients failed to complete this study due to treatment interruption and 4 requested withdrawal. Thus, only 437 cases with complete data were included in the analysis, with an effective recovery rate of 97%, all 437 samples included in the analysis were complete cases with no missing data, thus eliminating the need for missing data handling methods such as multiple imputation.

### 2.5 Research tools

#### 2.5.1 General information questionnaire

The research team developed a general information questionnaire based on a review of the literature and consultations with experts in gerontology, neurology, and nursing. The questionnaire includes items such as sex, age, lifestyle, physical exercise and language function (see Table 1). Low physical activity refers to less than three times per week, each time less than 30 min; high physical activity refers to three times or more per week, each time more than 30 min or longer. The judgment of language function was based on the patients’ medical records. Patients were categorized into the “Language Function—Abnormal” group if their records contained any notation of language impairment (e.g., “slurred speech,” “unclear articulation,” “dysarthria,” “verbal ambiguity”). Conversely, patients were classified into the “Language Function—Normal” group if records explicitly indicated intact language ability (e.g., “fluent speech,” “clear communication”).

**Table 1:** Comparison of social frailty scores in general characteristics of older adult patients with ischemic stroke (*n* = 437).

Variable	*n* (%)	SF score (*x* ± *s*)	*t/F*	*P*
Sex			−1.395^1^	0.164
Male	251 (57.4%)	1.94 ± 1.726		
Female	186 (42.6%)	2.16 ± 1.586		
Age, years			3.247^2^	0.021
60–69	135 (30.9%)	1.82 ± 1.714		
70–79	200 (45.8%)	1.97 ± 1.588		
80–89	91 (20.8%)	2.36 ± 1.683		
≥90	11 (2.5%)	3.00 ± 1.949		
Education			14.432^3^	<0.001
Primary school and below	287 (65.7%)	2.20 ± 1.636		
Middle school	95 (21.7%)	2.05 ± 1.765		
High school	44 (10.1%)	1.27 ± 1.500		
College and above	11 (2.5%)	0.64 ± 0.809		
Spousal status			−4.128^1^	<0.001
Have a spouse	325 (74.4%)	1.83 ± 1.598		
No spouse	112 (25.6%)	2.61 ± 1.747		
Monthly income, RMB			31.672^3^	<0.001
<1,000	217 (49.7%)	2.65 ± 1.589		
1,000–3,000	93 (21.3%)	1.90 ± 1.582		
3,001–5,000	72 (16.5%)	1.08 ± 1.361		
>5,000	55 (12.6%)	1.04 ± 1.387		
Residence			−4.620^1^	<0.001
Urban	236 (54%)	1.70 ± 1.645		
Rural	201 (46%)	2.42 ± 1.617		
Living arrangement			−4.176^1^	<0.001
Living alone	65 (14.9%)	2.82 ± 1.629		
Living with family	372 (85.1%)	1.90 ± 1.641		
Pre-retirement occupation			18.887^3^	<0.001
Worker	89 (20.4%)	1.55 ± 1.692		
Farmer	224 (51.3%)	2.48 ± 1.506		
Business/enterprise practitioner	88 (20.1%)	1.19 ± 1.477		
Others	36 (8.2%)	2.50 ± 1.949		
Type of health insurance			41.290^3^	<0.001
Employee health insurance	135 (30.9%)	1.10 ± 1.338		
Resident health insurance	296 (67.7%)	2.44 ± 1.644		
Others	6 (1.4%)	3.00 ± 1.095		
Religious belief status			0.913^1^	0.362
Yes	44 (10.1%)	2.25 ± 1.793		
No	393 (89.9%)	2.01 ± 1.656		
Use of smartphone/computer			−6.459^1^	<0.001
Yes	210 (48.1%)	1.52 ± 1.557		
No	227 (51.9%)	2.51 ± 1.633		
Stroke family history			0.154^1^	0.878
Yes	65 (14.9%)	2.06 ± 1.749		
No	372 (85.1%)	2.03 ± 1.658		
History of falls			1.793^1^	0.074
Yes	172 (39.4%)	2.21 ± 1.634		
No	265 (60.6%)	1.92 ± 1.686		
Times of strokes			−2.626^1^	0.009
First time	232 (53.1%)	1.84 ± 1.617		
Recurrence	205 (46.9%)	2.25 ± 1.705		
Types of prescribed medications			7.132^2^	<0.001
1–2	88 (20.1%)	1.44 ± 1.625		
3–4	196 (44.9%)	2.15 ± 1.618		
≥5	153 (35%)	2.22 ± 1.695		
Number of chronic diseases			2.801^2^	0.062
<3	299 (68.4%)	1.91 ± 1.621		
3–5	119 (27.2%)	2.34 ± 1.772		
>5	19 (4.3%)	2.05 ± 1.615		
Language function			−4.526^1^	<0.001
Normal	226 (51.7%)	1.69 ± 1.636		
Abnormal	211 (48.3%)	2.40 ± 1.631		
Physical activity			4.507^1^	<0.001
Low	316 (72.3%)	2.25 ± 1.663		
High	121 (27.7%)	1.46 ± 1.555		
Walking status			28.771^2^	<0.001
Walk independently	251 (57.4%)	1.59 ± 1.558		
Use a walking aid	137 (31.4%)	2.42 ± 1.621		
Unable to walk	49 (11.2%)	3.24 ± 1.507		
Duration of illness, years			3.889^2^	0.009
<1	243 (55.6%)	1.88 ± 1.580		
<3	34 (7.8%)	2.85 ± 1.794		
≤5	85 (19.5%)	1.98 ± 1.697		
>5	75 (17.2%)	2.23 ± 1.767		
Lesion location			4.127^3^	0.007
Left	130 (29.7%)	2.42 ± 1.817		
Right	137 (31.4%)	2.00 ± 1.706		
Bilateral	127 (29.1%)	1.79 ± 1.541		
Others	43 (9.8%)	1.67 ± 1.210		

^1^*t*-values; ^2^*F*-values; ^3^when the variance is not uniform, the Welch test was used.

#### 2.5.2 Help, Participation, Loneliness, Financial, Talk Scale (The HALFT scale)

This scale was developed by Chinese scholar Ma et al. (21) based on community-dwelling older adults in Beijing. It consists of five items:

1. Are you helpful to your family or friends?
2. Do you participate in any social or leisure activities?
3. Do you feel lonely?
4. Is your income sufficient to cover your daily expenses?
5. Do you have someone to talk with every day?

For items 1, 2, 4, and 5, a “Yes” response was scored as 0 point and a “No” response as 1 points. For item 3, a “Yes” response was scored as 1 point and a “No” response as 0 points. The total score ranges from 0 to 5, with scores ≥3 indicating the presence of social frailty; higher scores reflect greater levels of social frailty. This scale has been widely used in populations such as older adult stroke patients (16), older adult patients with heart failure (22), and older adult patients with chronic obstructive pulmonary disease (23) in China. The Cronbach’s *α* was 0.752 in this study.

#### 2.5.3 The Athens Insomnia Scale (AIS)

The AIS was utilized in this study to evaluate patients’ sleep quality. This scale was developed by Soldatos et al. (24) and it consists of eight items:

1. Time it takes you to fall asleep after turning-off the lights.
2. Awakenings during the night.
3. Final awakening earlier than desired.
4. Total sleep duration.
5. Overall quality of sleep (no matter how long you slept).
6. Sense of well-being during the day.
7. Functioning (physical and mental) during the day.
8. Sleepiness during the day.

A 4-point Likert scale is used for scoring, where responses range from “no problem” to “severely affected,” corresponding to scores of 0–3. The total score ranges from 0 to 24, with higher scores indicating poorer sleep quality. The Cronbach’s *α* was 0.863 in this study.

#### 2.5.4 Barthel Index Scale (BI)

The BI scale was used to assess patients’ ADL. This scale was developed by Hou et al. (25), and it includes 10 items:

1. Bowel Control: scored as 0 (incontinent or requires enema), 5 (occasional accidents or needs prompting), or 10 (full control).
2. Bladder Control: scored 0 (incontinent or indwelling catheter), 5 (occasional accidents), or 10 (complete control).
3. Grooming: scored 0 (dependent) or 5 (independent with or without aids).
4. Toilet Use: scored 0 (dependent), 5 (needs assistance), or 10 (independent).
5. Feeding: scored 0 (dependent on feeding tube or full assistance), 5 (needs help with cutting or setup), or 10 (independent).
6. Transfer: measures ability to move between bed and chair, scored 0 (unable), 5 (requires one person assistance), 10 (requires supervision or minimal aid), or 15 (independent).
7. Mobility: Assesses ambulation on level surfaces for at least 50 meters, scored 0 (immobile), 5 (wheelchair-independent), 10 (needs assistance to walk), or 15 (independent).
8. Dressing: scored 0 (dependent), 5 (needs partial help), or 10 (independent).
9. Stairs: measures ability to ascend and descend one flight of stairs, scored 0 (unable), 5 (needs assistance or supervision), or 10 (independent).
10. Bathing: scored 0 (dependent) or 5 (independent).

The total score ranges from 0 to 100, with higher scores indicating better ADL. The Cronbach’s *α* was 0.916 in the stroke patient population (25). The Cronbach’s α was 0.901 in this study.

#### 2.5.5 The 5-item version of the Geriatric Depression Scale (GDS-5)

The GDS-5 was developed by Hoyl et al. (26) as a simplified version of the GDS-15 that retains five core items:

1. Are you basically satisfied with your life?
2. Do you often get bored?
3. Do you often feel helpless?
4. Do you prefer to stay at home rather than go out and do new things?
5. Do you feel pretty worthless the way you are now?

For Item one, a “Yes” response is scored as 0 and a “No” response as one; the scoring rule is reversed for the remaining items. The total score ranges from 0 to 5, with higher scores indicating a more severe level of depression. In the depression screening of older adult populations, this scale has a sensitivity of 0.94, a specificity of 0.81, an inter-rater reliability of 0.88, and a test–retest reliability of 0.84 (27). The Cronbach’s *α* was 0.789 in this study.

#### 2.5.6 Social Support Rating Scale (SSRS)

The SSRS, developed by Xiao (28), was used in this study. It consists of 3 dimensions—subjective support, objective support, and support utilization—and a total of 10 items. The details are as follows:

Subjective Support comprises four items:

1. Number of close friends providing support (scored 1–4).
2. Closeness with neighbors (scored 1–4).
3. Level of concern from colleagues (scored 1–4).
4. Support and care from family members (scored 5–20).

Objective Support comprises three items:

1. Your living status in the past year (scored 1–4).
2. What were the sources of financial support or assistance for solving practical problems you received when encountering emergencies in the past? (scored 0–9).
3. What were the sources of comfort or care you received when encountering emergencies in the past? (scored 0–9).

Support utilization comprises three items:

1. Your way of venting when encountering troubles (scored 1–4).
2. Your way of seeking help when encountering troubles (scored 1–4).
3. Your frequency of participating in group organization activities (scored 1–4).

The total score ranges from 12 to 66, with higher scores indicating a higher level of social support. This scale has a test–retest reliability of 0.92 and demonstrates good validity (28). The Cronbach’s *α* was 0.863 in this study, the Cronbach’s α of subjective support, objective support and support utilization degree were 0.722, 0.703, and 0.777, respectively.

### 2.6 Data analysis

The research data were entered into Microsoft Excel 16.0 (Microsoft Corporation, Redmond, Washington, United States) and verified by double entry. Statistical analyses were performed using IBM SPSS 27.0 (IBM Corporation, Armonk, State of New York, United States). Continuous data were tested for normality using P–P plots and were found to be normally distributed, thus being described as mean ± standard deviation (x̄±s). Categorical data were summarized with frequencies and percentages. Common method bias was tested using Harman’s single-factor test. Univariate analyses were performed to identify potential factors associated with social frailty. For this, independent samples *t*-tests were used for two-group comparisons, and one-way ANOVA was applied for comparisons across multiple groups. Pearson correlation analysis was employed to examine the relationships between continuous independent variables and social frailty. Variables that reached a significance level of *p* < 0.05 in univariate and correlation analyses were considered candidate variables for inclusion in the multivariate model. Multivariate analysis was conducted using multiple linear regression. A *p* < 0.05 was considered statistically significant.

Structural equation modeling was performed with IBM SPSS Amos 26.0 (IBM Corporation, Armonk, State of New York, United States). The maximum likelihood method was employed for parameter estimation and direct path testing. The model was considered to have a good fit when χ^2^/df (Chi-square/Degrees of Freedom) <3, RMSEA (Root Mean Square Error of Approximation) <0.08, CFI (Comparative Fit Index) >0.9, NFI (Normed Fit Index) >0.9, and TLI (Tucker-Lewis Index) >0.9. The mediating effect was tested using the Bootstrap method with 5,000 resamples. A mediating effect was considered to exist if the 95% confidence interval did not contain zero.

## 3 Results

### 3.1 Common method bias test

The common method bias test was carried out by Harman single-factor test. In this study, the characteristic root of 10 factors was >1, and the variance explained rate of the first factor was 23.063%, which was <40% of the critical standard, indicating that there was no common method bias in this study (29).

### 3.2 Participants’ characteristics

In this study, 251 cases were male (57.4%) and 186 cases were female (42.6%); 232 cases were first stroke (53.1%), and 205 cases experienced recurrent stroke (46.9%) (see Table 1 for other conditions).

### 3.3 Scores of various scales

The HALFT score of older adult patients with ischemic stroke was 2.03 ± 1.67 [cutoff for social frailty: ≥3 (21)], the GDS-5 score was 1.62 ± 1.65 [cutoff for depression: ≥2 (26)], the AIS score was 6.14 ± 4.705 [cutoff for insomnia: ≥7 (24)], the BI score was 74.12 ± 21.72 [indicating mild dependence, cutoff: 61–99 (25)], and the SSRS score was 34.88 ± 7.313 [indicating moderate social support, cutoff: 23–44 (28)]. The scores of each dimension of the SSRS scale were subjective support 19.65 ± 3.691, objective support 8.72 ± 2.656 and support utilization 6.51 ± 2.695.

### 3.4 Univariate analysis

The social frailty scores of older adult patients with ischemic stroke showed statistically significant differences across variables such as age, education level, spousal status, personal monthly income, place of residence, living style, pre-retirement occupation type, medical insurance type, use of smartphone/computer, times of strokes, types of medication, language function, physical activity, walking status, duration of illness, and lesion location (*p* < 0.05), as shown in Table 1.

### 3.5 Correlation analysis

The correlation analysis showed that social frailty was significantly positively correlated with sleep quality (*r* = 0.277, *p* < 0.01), depression (*r* = 0.495, *p* < 0.01), and negatively correlated with ADL (*r* = −0.384, *p* < 0.01) and social support (*r* = −0.523, *p* < 0.01).

### 3.6 Results of multivariate stepwise linear regression analysis

Taking social frailty as the dependent variable, a multiple stepwise linear regression analysis was conducted using variables that were statistically significant in univariate and correlation analyses as independent variables. The assignment of independent variables is presented in Table 2. The results revealed that employee health insurance, impaired language function, recurrent stroke, social support, depression, ADL, and sleep quality were significant factors affecting social frailty in older adult patients with ischemic stroke. These factors collectively accounted for 45.9% of the total variance (see Table 3 for details).

**Table 2:** Independent variable assignment methods.

Variable	Assignment
Age, years	60–69 = 1; 70–79 = 2; 80–89 = 3; ≥90 = 4.
Education	Primary school and below = 1; Middle school = 2; High school = 3; College and above = 4.
Spousal status	Have a spousal = 1; No spousal = 2.
Residence	Urban = 1; Rural = 2.
Living arrangement	Living with family = 1; Living alone = 2.
Monthly income, RMB	<1,000 = (0,0,0,0); 1,000–3,000 = (0,1,0,0); 3,001–5,000 = (0,0,1,0); >5,000 = (0,0,0,1).
Pre-retirement occupation	Farmer = (0,0,0,0); Worker = (0,1,0,0); Business/enterprise practitioner = (0,0,1,0); Others = (0,0,0,1).
Type of health insurance	Resident health insurance = (0,0,0); Employee health insurance = (0,1,0); Others = (0,0,1).
Use of smartphone/computer	No = 1, Yes = 2.
Types of prescribed medications	1–2 = (0,0,0); 3–4 = (0,1,0); ≥5 = (0,0,1).
Times of strokes	First = 1; Recurrence = 2.
Language function	Normal = 1; Abnormal = 2.
Physical activity	Low = 1; High = 2.
Walking status	Walk independently = (0,0,0); Use a walking aid = (0,1,0); Unable to walk = (0,0,1).
Duration of illness, years	<1 = (0,0,0,0); <3 = (0,1,0,0); <5 = (0,0,1,0); >5 = (0,0,0,1).
Lesion location	Left = (0,0,0,0); Right = (0,1,0,0); Bilateral = (0,0,1,0); Others = (0,0,0,1).

**Table 3:** Multiple linear regression analysis of influencing factors of social frailty in older adult patients with ischemic stroke (*n* = 437).

Variable	*B*	*SE*	*β*	*t*	*P*	Correlation statistics	
						Tolerance	*VIF*
(Constant)	4.265	0.465	-	9.162	<0.001	–	–
Sleep quality	0.03	0.014	0.085	2.186	0.029	0.83	1.205
ADL	−0.015	0.003	−0.192	−5.081	<0.001	0.867	1.153
Depression	0.234	0.043	0.231	5.473	<0.001	0.695	1.438
Social support	−0.067	0.009	−0.293	−7.348	< 0.001	0.778	1.285
Employee health insurance	−0.71	0.137	−0.197	−5.162	<0.001	0.855	1.169
Abnormal language function	0.288	0.121	0.086	2.37	0.018	0.938	1.066
Stroke recurrence	0.29	0.12	0.087	2.428	0.016	0.97	1.031

*R* = 0.684, *R*^2^ = 0.468, adjusted *R*^2^ = 0.459, *F* = 53.892, Durbin-Watson test = 1.699, *p* < 0.001. ADL, activities of daily living.

### 3.7 Structural equation model

#### 3.7.1 Model construction of factors affecting social frailty in older adult patients with ischemic stroke

According to the research hypothesis, a structural equation model was constructed with ADL, sleep quality, and depression as independent variables, social frailty as the dependent variable, and social support as the mediating variable. Because both the AIS scale (assessing sleep quality) and the BI scale (assessing ADL) are unidimensional with multiple items, item parceling was employed to group items from these two latent variables into parcels, which served as measurement variables to better analyze the relationships between latent variables (30, 31).

**Figure 2:**
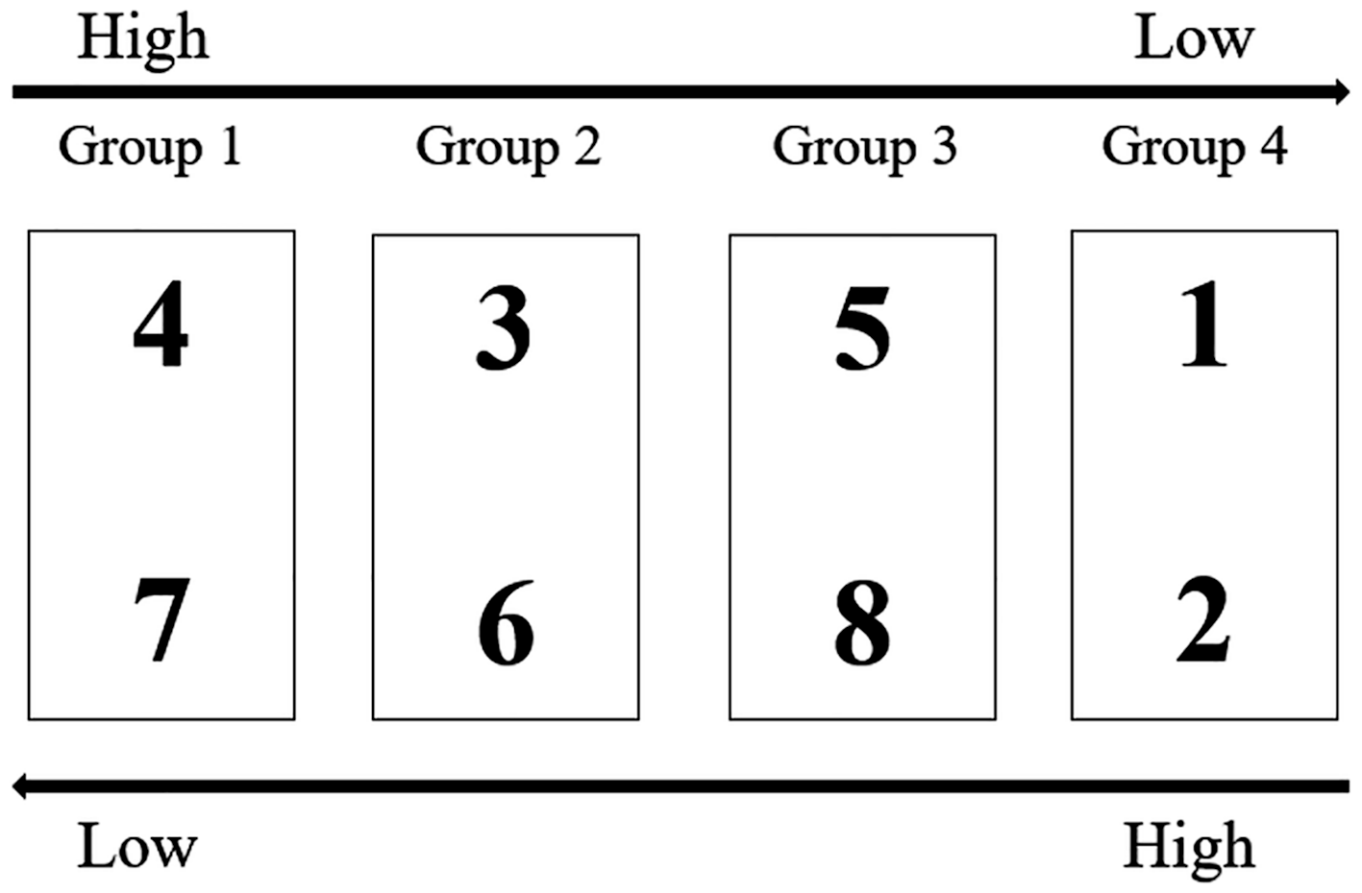
Factor balancing method.

The “factor balancing method” was used for parceling (31). Taking the AIS scale (8 items) as an example, items were ranked by factor loadings in descending order: Item 4, 3, 5, 1, 2, 8, 6, 7. The first four items were paired with the last four items in reverse order, resulting in 4 groups (see Figure 2): Group 1 (Items 4 and 7), Group 2 (Items 3 and 6), Group 3 (Items 5 and 8), Group 4 (Items 1 and 2).

The same method was applied to parcel the BI scale items.

**Figure 3:**
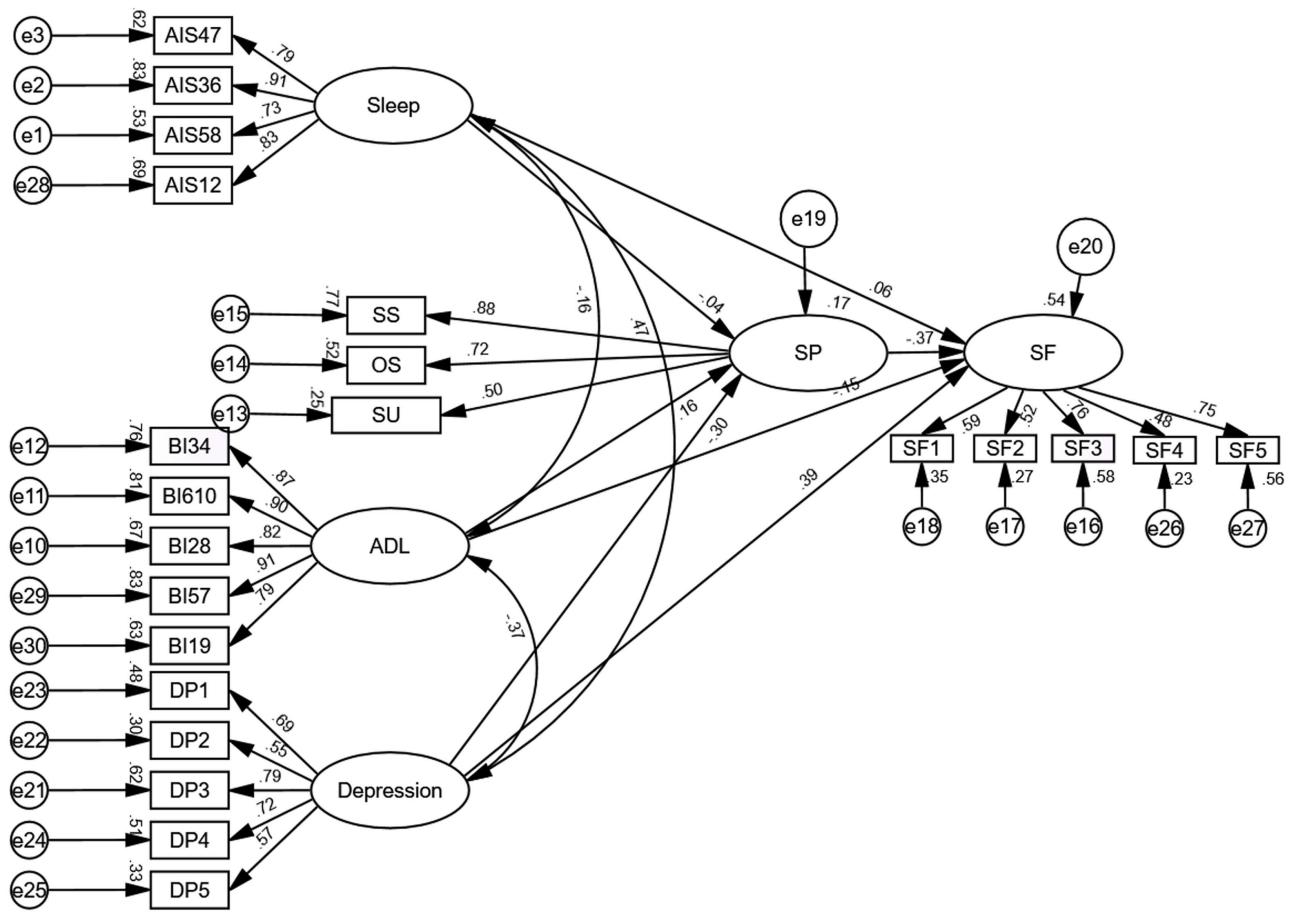
Structural equation model of influencing factors of social frailty in older adult patients with ischemic stroke (standardized). (1) SP: Social support, SS: Subjective support, OS: Objective support, SU: Support utilization; SF: Social frailty; (2) The naming convention for measuring variables related to Sleep and ADL is as follows: scale abbreviation + packaged items.

The model was fitted using the maximum likelihood method. The results showed:

χ^2^/ν = 2.809 (<3), RMSEA = 0.064 (<0.08), NFI = 0.909 (>0.9), IFI = 0.927 (>0.9), TLI = 0.915 (>0.9), CFI = 0.927 (>0.9), indicating a good model fit. The model is shown in Figure 3.

#### 3.7.2 Direct path analysis of factors affecting social frailty in older adult patients with ischemic stroke

Path analysis indicated that in the physiological dimension, although the direct effect of sleep quality accounted for 6% of the total direct effect on social frailty, this association was not statistically significant (*β* = 0.057, *p* = 0.282). Conversely, ADL showed a statistically significant direct negative effect on social frailty (*β* = −0.154, *p* = 0.002), representing 16% of the total direct effect.

In the psychological dimension, depression demonstrated a significant direct positive effect on social frailty (*β* = 0.390, *p* < 0.001), accounting for 40% of the total direct effect.

Within the social dimension, social support demonstrated a significant direct negative effect on social frailty (*β* = −0.373, *p* < 0.001), accounting for 38% of the total direct effect.

Collectively, depression demonstrated the largest effect size on social frailty through direct pathways, followed by social support, activities of daily living, and sleep quality, in descending order of magnitude (see Table 4).

**Table 4:** Direct path analysis of factors influencing social frailty in older adult patients with ischemic stroke.

Direct path	*β*	*S.E.*	*P*	Quantity of effect
Sleep quality → Social frailty	0.057	0.023	0.282	6%
ADL → Social frailty	−0.154	0.006	0.002	16%
Depression → Social frailty	0.390	0.064	<0.001	40%
Social support → Social frailty	−0.373	0.050	<0.001	38%

ADL, activities of daily living.

#### 3.7.3 Mediation analysis of factors contributing to social frailty in older adult patients with ischemic stroke

A bootstrap resampling procedure with 5,000 iterations was employed to examine the mediating effects. The results indicated that, in the physiological dimension, a significant mediation effect of social support was observed between ADL and social frailty (*β* = −0.061, *p* = 0.003, 95% CI: −0.114 to −0.019), with the indirect effect accounting for 28% of the total effect in the ADL-social frailty pathway. In contrast, no significant mediating role was found for social support in the relationship between sleep quality and social frailty (*β* = 0.014, *p* = 0.546, 95% CI: −0.032 to 0.062).

In the psychological dimension, a significant mediation effect of social support was observed between depression and social frailty (*β* = 0.113, *p* < 0.001, 95% CI: 0.063–0.181), with the indirect effect accounting for 22% of the total effect in the depression-social frailty pathway (see Table 5).

**Table 5:** Mediator test of influencing factors of social frailty in older adult patients with ischemic stroke.

Model path	Effect	*β*	*S. E.*	*P*	95% CI	Quantity of effect
X1 → M → Y	Total effect	0.071	0.058	0.227	−0.046 ~ 0.186	100%
	Direct effect	0.057	0.056	0.289	−0.053 ~ 0.168	80%
	Indirect effect	0.014	0.024	0.546	−0.032 ~ 0.062	20%
X2 → M → Y	Total effect	−0.215	0.057	<0.001	−0.331 ~ −0.105	100%
	Direct effect	−0.154	0.054	0.004	−0.264 ~ −0.052	72%
	Indirect effect	−0.061	0.024	0.003	−0.114 ~ −0.019	28%
X3 → M → Y	Total effect	0.504	0.067	<0.001	0.368 ~ 0.631	100%
	Direct effect	0.390	0.070	0.001	0.247 ~ 0.525	78%
	Indirect effect	0.113	0.030	<0.001	0.063 ~ 0.181	22%

X1, sleep quality; X2, ADL, activities of daily living; X3, depression; M, social support; Y, social frailty.

The research hypothesis of this study is partially true (see Table 6).

**Table 6:** Hypothesis validation of the influencing factors of social frailty in older adult patients with ischemic stroke.

Dimensions	Research hypothesis	Result
Biological	ADL may directly affect social frailty.	True
	ADL can indirectly affect social frailty through social support.	True
	Sleep quality can directly affect social frailty.	False
	Sleep quality can indirectly affect social frailty through social support.	False
Psychological	Depression can directly affect social frailty.	True
	Depression can indirectly affect social frailty through social support.	True
Social	Social support can directly affect social frailty.	True

ADL, activities of daily living.

## 4 Discussion

### 4.1 The prevalence of social frailty is relatively high in older adult patients with ischemic stroke

The results of this study showed that the social frailty score of older adult patients with ischemic stroke was (2.03 ± 1.67), among whom 167 people scored more than three, and the prevalence of social frailty was 38%, higher than that of older adults in Chinese communities (29.83%) (32). The reason may be that older adults with ischemic stroke often experience symptoms such as dizziness and decreased lower limb muscle strength after the stroke, which are associated with patients’ reduced activity range (to prevent falls) (33). Meanwhile, some patients require long-term care due to restricted physical mobility. The decline in self-management capacity passively weakens their social roles (e.g., as caregivers for grandchildren or community volunteers), further reducing social participation. In addition, long-term rehabilitation and medication impose substantial financial burdens, which may further diminish their material resources. The interplay of these factors collectively increases the risk of social frailty.

### 4.2 The influence path of social frailty in older adult patients with ischemic stroke

#### 4.2.1 The impact of ADL and sleep quality (biological dimension) on the level of social frailty in older adult patients with ischemic stroke

The results of this study showed that ADL had a negative predictive effect on the level of social frailty in patients. Decline or loss of ADL directly impairs patients’ ability to actively participate in social activities, increases spatial limitations on social interactions, and hampers emotional communication with the outside world, thereby narrowing the scope of social engagement (34, 35). These factors subsequently elevate the risk of social frailty. Additionally, ADL can indirectly affect patients’ social frailty level through social support. This indicates that improving patients’ social support level can buffer the adverse impact of impaired ADL on social frailty, which is consistent with findings reported by Wang et al. in heart failure patients (22). A decline in ADL is associated with decreased self-management capacity and increased caregiving burden. However, providing social support through means such as daily life care and material assistance can compensate for some of these harms and reduce the risk of social frailty.

Based on the above findings, a comprehensive intervention strategy can be implemented to address the risk of social frailty associated with declines in ADL and inadequate social support. Healthcare professionals should encourage and train patients to use digital technologies (such as social networking platforms and video communication software) to maintain social connections (36), thereby compensating for the social limitations brought about by restricted physical activity. In addition, healthcare institutions can develop personalized rehabilitation programs and provide relevant health education to encourage long-term patient adherence, enhancing physical activity levels and autonomy, which may alleviate social frailty. For instance, Gen et al. (37) conducted an 8-week randomized controlled trial of a multicomponent intervention targeting community-dwelling older adults with social frailty. Centered on health education and exercise, the intervention included walking training, stretching exercises, and body-weight training. Results showed that the program significantly reduced loneliness among older adults, which may positively impact social frailty.

Interestingly, the results of multiple linear regression analysis indicated that sleep quality was an influencing factor for social frailty in older adult patients with ischemic stroke (*p* = 0.029). This analysis assumes that the variables are relatively independent and have linear relationships, thereby reflecting the solitary effect of sleep quality on social frailty after controlling for other variables. However, in the structural equation model, this pathway was no longer statistically significant (*p* = 0.289). A possible explanation is that structural equation model considers complex relationships involving multiple variables and pathways; factors such as depression, social support, and ADL may jointly influence both sleep quality and social frailty, thus attenuating the direct association between sleep quality and social frailty. Nakakubo et al. (38) conducted a study involving 4,427 community-dwelling older adults, and the results indicated that sleep duration of ≥9 h (OR = 1.46, 95% CI: 1.14–1.84) and excessive daytime sleepiness (HR = 1.32, 95% CI: 1.02–1.71) were associated with an increased risk of social frailty. Conversely, another cross-sectional study involving 5,782 middle-aged and older adult community residents found no association between sleep quality, daytime sleepiness, or sleep duration and social frailty in both middle-aged and older groups (14). This suggests that there is currently no consensus on the relationship between sleep parameters and social frailty.

Furthermore, this study did not find that social support mediated the relationship between sleep quality and social frailty, which may be due to the limitations inherent in cross-sectional designs. The processes of social support improving sleep quality and sleep quality alleviating social frailty may likely long-term and dynamic; cross-sectional studies can only capture relationships at a single time point and cannot establish a chain effect such as “accumulation of social support → improvement in sleep quality → reduction in social frailty.” Future longitudinal studies are needed to further verify the relationships among these variables.

#### 4.2.2 The impact of depression (psychological dimension) on the level of social frailty in older adult patients with ischemic stroke

This study found that depression had a direct positive predictive effect on social frailty, indicating that higher levels of depression are associated with a greater likelihood of experiencing social frailty, which was consistent with the findings reported by Cataltepe et al. (39). Older adult ischemic stroke patients with depression often endure low mood, diminished interest in social activities, and social withdrawal, which are related to decreased social engagement (40). In turn, social frailty can negatively impact patients’ sense of happiness and may exacerbate depressive symptoms (41), creating a vicious cycle. Moreover, depression can indirectly influence social frailty through social support. This suggests that a lack of social support is a significant pathway through which depression transforms into social frailty. Studies have shown that stroke patients desire social support from healthcare providers, including the provision of disease-related knowledge and rehabilitation exercise recommendations (42). This type of support may help improve patients’ depressive symptoms and reduce the level of social frailty.

Given this mechanism, early identification and intervention of psychological symptoms, as well as enhancing social support, are of vital importance. In addition to the routine and dynamic monitoring of depressive symptoms, it may be necessary to implement certain interventions. For instance, healthcare professionals can establish peer support groups, enabling patients with similar experiences to communicate with each other. This can not only enhance emotional regulation abilities but also promote social connections and participation (43). Furthermore, the study by Jesus et al. (44) provides evidence for a relevant approach: the Homebound Elderly People Psychotherapeutic Intervention (HEPPI). Their research involved older adults with mild cognitive impairment and anxiety or depressive symptoms, who participated in this 10-week program. The program included psychoeducation, mindfulness-based stress reduction, behavioral activation, among other components. Results demonstrated that the intervention not only significantly reduced participants’ loneliness but also revealed that decreased social loneliness—a dimension of loneliness—acted as a significant mediator for improvements in depressive symptoms. Given that older adult patients with ischemic stroke also experience depression and loneliness, this approach may hold potential applicability for improving their psychosocial functioning. Therefore, healthcare providers and community workers could consider adapting the HEPPI model to develop and pilot similar programs specifically designed for homebound older adult stroke patients, with the aim of evaluating its effects on reducing loneliness, alleviating depressive symptoms, and ultimately decreasing social frailty.

#### 4.2.3 The impact of social support (social dimension) on the level of social frailty in older adult patients with ischemic stroke

This study showed that social support has a direct negative predictive effect on social frailty in older adult patients with ischemic stroke, the higher the level of social support, the lower the level of social frailty, which is consistent with Wu et al.’s report on the impact of social support on social withdrawal in stroke patients (45). Patients with high levels of social support typically receive more care, assistance, and social resources, compared to those with low support. Their social networks are richer, especially adequate family support can significantly improve patients’ quality of life in social domains (46). Therefore, family members should enhance family adaptability, create a positive family atmosphere, and assist patients in actively participating in social activities.

On the other hand, self-management ability, as one of the core concepts of social frailty, is also closely related to the level of social support. Studies have shown that patients with higher levels of social support are more likely to demonstrate better self-management behaviors (47), possibly because robust social support provides not only tangible resources such as rehabilitation guidelines, medication information, financial security but also emotional support such as companionship, treatment supervision. These combined supports facilitate more effective self-management among patients (48, 49).

Additionally, adequate social support from family members, friends, healthcare professionals, and other sources is conducive to helping older adult patients regain their social roles, reintegrate into society, and improve their quality of life (50). Therefore, it is recommended to establish a “family, community, and hospital” integrated support system to fully mobilize patients’ social support and resources, and to prevent social frailty.

### 4.3 Other influencing factors of social frailty in older adult patients with ischemic stroke

The results of multiple linear regression analysis showed that employee health insurance, impaired language function, and stroke recurrence were also influencing factors for social frailty in patients.

Compared to patients with resident health insurance, those with employee health insurance had a lower risk of experiencing social frailty. This may be because, in China, the reimbursement rate for inpatient expenses is higher for patients with employee health insurance than for those with resident health insurance, thereby reducing their financial burden from inpatient treatment and providing access to more medical resources such as expensive medications, advanced medical equipment, and sophisticated treatment modalities, which may be associated with a reduced risk of social frailty.

The results of this study showed that patients with impaired language function were more likely to experience social frailty than those with normal language function. Patients with impaired language function may experience difficulties in communicating with others (51), which may be associated with decreased sense of social experience as well as increased rates of social isolation and alienation. It is recommended that healthcare professionals implement group conversation treatment for this patient population. This approach has been shown to significantly improve language communication abilities in patients with chronic aphasia (52, 53), therefore, its potential role in addressing social frailty warrants further investigation.

Moreover, patients who have had a recurrence of stroke are more likely to experience social frailty. Recurrence of stroke may be associated with a decrease in psychological resilience, which may make patients more likely to have a negative outlook on rehabilitation exercises and lose confidence in their recovery (54). This consequently reduces their enthusiasm for reintegration into society. On the other hand, they may also face more severe physical impairments, limiting their ability to fulfill social roles (e.g., family caregiving) (55), thus increasing their susceptibility to social frailty. Healthcare providers can set up patient activity centers or group rehabilitation centers within departments, promoting interaction among patients through organized group entertainment activities or group rehabilitation exercises, thereby reducing feelings of loneliness and levels of social frailty.

### 4.4 Limitations of the study

(1) As this study was a cross-sectional study and only surveyed inpatients admitted to the neurology departments of two hospitals in Sichuan Province, China, it could not explore causal relationships or changing trends among variables, and was subject to certain geographical limitations. In the future, multi-center longitudinal studies could be conducted to promote field development. (2) Several important covariates, such as stroke severity/NIHSS, time since index stroke, comorbidity burden, were not adjusted for in the analysis. Future studies should aim to include these variables for a more comprehensive adjustment. (3) Our reliance on clinician descriptions in electronic medical records to determine language function in this study, without corroboration with standardized objective scales, is a potential source of bias. Future studies should employ validated objective tools to improve assessment accuracy and confirm the robustness of these conclusions.

## 5 Conclusion

The level of social frailty in older adult patients with ischemic stroke is influenced by multiple factors. Healthcare providers should focus on patients with low levels of social support, depression, decreased ADL, low health insurance reimbursement rates, recurrent strokes, and impaired language function. In particular, targeted interventions aimed at improving social support levels and alleviating depressive symptoms to have significant implications for reducing the level of social frailty in patients.

## Data Availability

The datasets presented in this article are not readily available because the datasets used and analyzed during the current study available from the corresponding author on reasonable request. Requests to access the datasets should be directed to Li Li, lili850249381@163.com.
